# Supplementary material for: Understanding crystallization pathways leading to manganese oxide polymorph formation
Source: Nat Commun. 2018 Jun 29;9:2553. doi: 10.1038/s41467-018-04917-y (PMC6026189; doi:10.1038/s41467-018-04917-y)
Supplement: Supplementary file 1 — Supplementary Information [file 41467_2018_4917_MOESM1_ESM.pdf]

Supplementary Information

Understanding Crystallization Pathways Leading to Manganese Oxide Polymorph Formation

Chen et al.

Supplementary Note 1. pH and oxidation reduction potential measurements.

pH and oxidation reduction potential (ORP) of the solution before and after the ex-situ runs were measured by a pH/ORP meter (VWR Traceable). The pH and ORP data are listed in Supplementary Table 1. The pH does not change significantly during the hydrothermal reaction, while the ORP decreased from ~1200 mV to ~ 400 mV. The reason is that the precipitation of metal oxides consumes oxygen from the aqueous solution, which is buffered by the water-oxygen equilibrium: $H_2O \rightleftarrows 1/2\ O_2 + 2H^+ + 2e^-$. This reduces the solution redox potential during crystal growth of MnO_2. Although this will influence the free-energy of the MnO_4^- precursor, it does not affect the relative free-energies between solid phases with the same oxygen content per formula unit (i.e., between different MnO_2 polymorphs, or between α-$K_{0.125}MnO_2$ and δ-$K_{0.33}MnO_2$). This means that the free-energy landscape of the solid phases in the K-Mn-H_2O system, as shown in Figure 2 in the main text, does not depend on the redox potential.

Supplementary Table 1. pH and ORP values before and after ex-situ hydrothermal reaction.

	**Before reaction**		**After reaction**	
	**pH**	**ORP (mV)**	**pH**	**ORP (mV)**
$P_{K=0.33}$	0.95	1210	2^*	495
$P_{K=0.2}$	1.00	1199	1.75	415
$P_{K=0}$	1.40	1189	1.99	422

* Measured by pH paper

Supplementary Note 2. **Basic Crystallographic Parameters of the MnO_2 phases**.

Supplementary Table 2. Detailed information for all the MnO_2 phases observed in this article. The related .cif files are available online as Supplementary Data.

Phase name	Space group	Crystallographic parameters		Notes	References
		(a, b, c) (Å)	(α, β, γ) (º)		
α	I4/m	a = 9.793<br>b = 9.793<br>c = 2.841	α = 90<br>β = 90<br>γ = 90	Previously known phase, with (2 × 2) tunnel structure.	Post 1982 [1]
β	$P4_2$/mnm	a = 4.411<br>b = 4.411<br>c = 2.878	α = 90<br>β = 90<br>γ = 90	Previously known phase, with (1 × 1) tunnel structure.	Baur 1976 [2]
δ	-	-	-	Previously known *family* of layered phases. In this work, two variations, δ' and δ", are observed.	Post 1999 [3]
δ'	Pma2	a = 14.000<br>b = 4.913<br>c = 8.568	α = 90<br>β = 90<br>γ = 90	Layered phase with K^+ intercalation between the layers, observed along $P_{K = 0.2}$ reaction pathway.	Post 1999 [3]
δ"	-	-	-	*Disordered*, platelet-like phase observed along $P_{K=0}$ reaction pathway.	Giovanoli 1980 [4]<br>Brock 1999 [5]
γ'	-	-	-	Previously known *disordered* phase *family* with (1 × 1) tunnel randomly grown into the (1 × 2) tunnel structure, observed along $P_{K=0}$ reaction pathway.	de Wolff 1959 [6]<br>Chabre 1995 [7]
R	Pnma	a = 14.036<br>b = 2.933<br>c = 4.467	α = 90<br>β = 90<br>γ = 90	Previously known phase with pure (1 × 2) tunnel structure.	de Wolff 1959 [6]<br>Chabre 1995 [7]<br>Kitchaev 2016 [8]
γ	C2/m	a = 9.395<br>b = 2.932<br>c = 4.646	α = 90<br>β = 90<br>γ = 90	Idealized phase with an ordered 50/50 intergrowth between R and β.	Kitchaev 2016 [8]

Supplementary Note 3. ***In-situ*** **wide-angle X-ray (WAXS) scattering measurements.**
WAXS data were collected at Beamline 11-3 at the Stanford Synchrotron Radiation Lightsource (SSRL) with an incident wavelength of 0.9744 Å. Experimental data were collected by using a Rayonics 225 2D detector. The data is collected over 0.56 Å$^{-1} \le q \le 4.92$ Å^{-1}, where $q = \frac{4\pi}{\lambda}\sin\theta$, where λ is the incident wave length and θ is the diffraction angle. The sample-to-detector distance was approximately 200 mm, which was calibrated by a 325-mesh Si powder standard.

A capillary tube (Kimble, 70 mm in length/1 mm in diameter) was used as the hydrothermal reactor. A custom-build sample holder/heater, as shown in Supplementary Figure 1, was used to heat up the reactors. The heat is applied to the reactor by using an Omega Lux air heater. An external variac controlled the temperature of the reactor. To protect the 2D detector in the event of capillary failure during the reaction, the sample holder was covered by a layer of Kapton film. To avoid sudden increase or decrease of scattering intensity due to random movements of crystalline MnO_2 and air bubbles in the reactor, the reactor was translated in its longitudinal direction for ± 1 mm during the measurements.

During the hydrothermal reaction, WAXS data were collected consecutively with a 1 min exposure time. The WAXS profiles were acquired by integrating individual 2D scattering patterns over 30 $^{o} \leq \chi \leq 150^{o}$, where χ is the polar angle. The intensity of WAXS profiles were normalized by the intensity of the incident beam measured by an ion chamber upstream of the sample. The backround of the WAXS profile was partially removed by under subtracting the profile at t = 0. Note that the incomplete removal of the background is caused by the variation of capillary wall thickness because of the ± 1 mm translation, and variation of the composition in the aqueous phase due to the formation of MnO_2. Therefore, the profile at t = 0 was multiplied with a constant less than 1 before subtraction to avoid over-subtraction.

The phase evolution of the three reaction pathways investigated in this work all completed within the first 90 min during the reaction and the end phase remain stable for up to 24 hr of additional reaction time. Supplementary Figure 2 compares the WAXS profiles at 90 min and 24 hr.

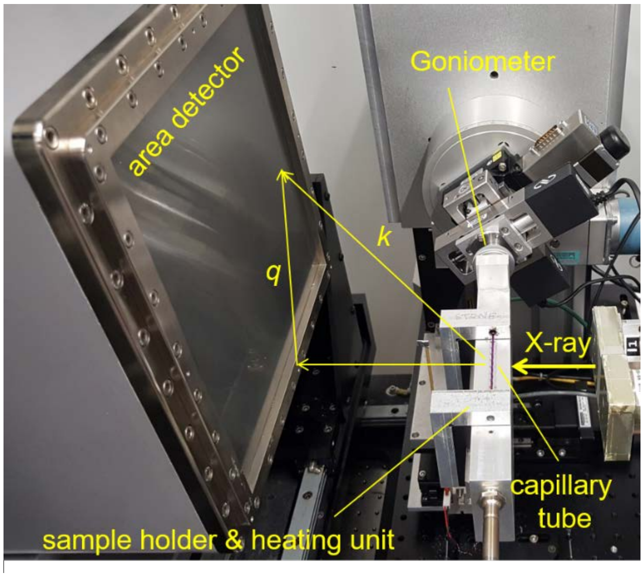

Supplementary Figure 1. The set-up of in-situ hydrothermal reaction at SSRL beamline 11-3 in this work. Different parts of the instruments are marked accordingly.

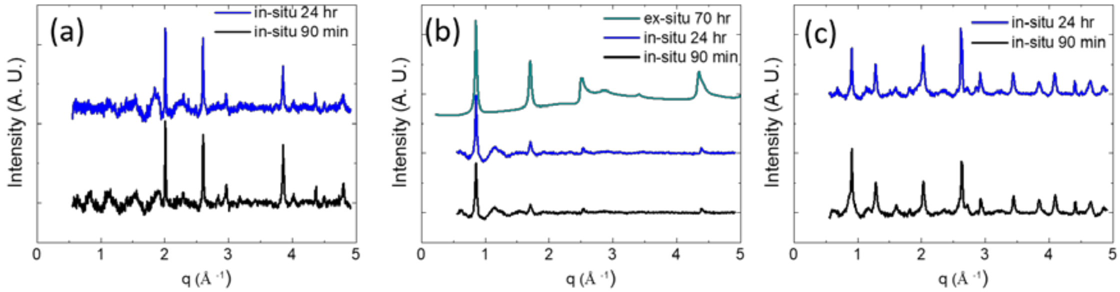

Supplementary Figure 2. *In-situ* WAXS profile at 90 min and 24 hr of (a) $P_{K=0}$, (b) $P_{K=0.33}$, and (c) $P_{K=0.2}$, showing the end phases are β, δ, and α- MnO_2, respectively. For $P_{K=0.33}$, the δ phase is stabilized even after 70 hr of ex-situ reaction, as marked in (b). The background of the in-situ profiles were manually removed by using GSAS-II software.

Supplementary Note 4. Phase fraction evolution during hydrothermal synthesis.
When phase x and y co-exist, the fraction of phase *x* (f) can be calculated from their scattering volumes (V) by using:

$$f_x = \frac{V_x}{V_x + V_y} \;\ldots\ldots(1)$$

In the equation above, V_x is related to the intensity of *hkl* diffraction peaks (I_{hkl}) of phase *x* by [9]:

$$I_{hkl} \propto I_0 \cdot |F_{hkl}|^2 \cdot m_{hkl} \cdot L(\theta) \cdot P(\theta) \cdot \exp(-B\, sin^2\theta/\lambda^2) \cdot V_x \;\ldots\ldots(2)$$

, where I_0 is the intently of the incident X-ray, F_{hkl} is the scattering factor of phase x, m_{hkl} is the multiplicity, $L(\theta) = \frac{1}{\sin(\theta)^2 \cos(\theta)}$ is the Lorentz factor, $P(\theta)$ is the polarization factor, and $\exp(-B\, sin^2\theta/\lambda^2)$ is the temperature factor. Here, $P(\theta)$ is approximated by 1 because of the horizontal polarization in synchrotron radiation and the small scattering angles. We also assume the temperature factor to be a constant because $\exp(-B\, sin^2\theta/\lambda^2)$ is not a strong function of the relatively narrow θ range that we are observing. When calculating I_{hkl}, symmetrical diffraction peaks can be fitted by a Lorentzian with linear background:

$$L(x) = C \cdot \frac{(\frac{w}{2})^2}{(x - x1)^2 + (\frac{w}{2})^2} + Bx + A \;\ldots\ldots(3)$$

, where *w* is the full width half maxima, *x1* is the position of the peak, and A, B, and C are constants. The area under a Lorentzian peak can be calculated by using[10]: $Area = \frac{\pi}{2} \cdot w \cdot C$. For peak shapes that are impacted by stacking faults, such as q = 2.58, and 4.45 $Å^{-1}$ for the δ' phase, the line shape is relatively more complicated and difficult to be fitted by a simple equation. Therefore, the area under the peak was obtained by summing up the counts under the peak, following by subtracting the right trapezoid-shaped background area (assuming a linear background) from the sum, as shown in Supplementary Figure 3.

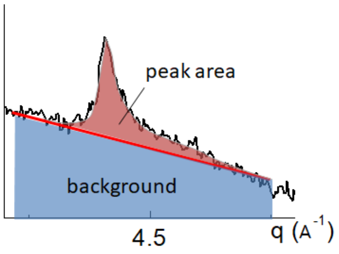

Supplementary Figure 3. An example showing how the background was subtracted for asymmetric diffraction peaks. The area shaded in red and the right trapezoid-shaped in blue represent the peak area and background, respectively.

Supplementary Note 5. Rietveld refinement of the end MnOx phases.

The Rietveld refinement parameters of the α and β MnO_2 phases were obtained by using GSAS-II software package[11] and are listed in Supplementary Table 3.

Supplementary Table 3. Full refinement parameters of the α and β MnO_2.

**Phase name**	**α-MnO_2**	**β -MnO_2**
Space group	I4/m	P42/mnm
Temperature	25 °C	25 °C
**Unit cell dimensions**		
a (Å)	9.7934	4.4108
b (Å)	-	-
c (Å)	2.8408	2.8781
λ (Å)	0.9744	0.9744
R_w	1.008	1.596
**Atomic Positions**		

	atom	x	y	z	frac.	atom	x	y	z	frac.
	Mn1	0.150	0.332	0.500	1.00	Mn1	0	0	0.500	1.00
	O2	0.000	0.653	0.500	1.00	O1	0.222	0.222	0	1.00
	O3	0.143	0.205	0.000	1.00					
	K4	0.500	0.500	0.000	0.10					

Supplementary Note 6. Calculating the fraction of (1 × 1) intergrowth in the γ' phase.

The fraction of (1 × 1) pyrolusite intergrowth (P_y) in the γ'-MnO_2 phase can be determined by using Chabre et al's[7] metric of the (1,3,0) (q = 2.56 Å^{-1}) diffraction peak. Compared to a defectless R-phase, the (1,3,0) peak of the γ'-MnO_2 phase is shifted by δb^*, which is used to calculate P_y by using the equation $P_r = 3\delta - 10.7\,\delta^2 + 18.5\,\delta^3$. In our case, the value of P_y of is calculated to be ~ 0.3, which remains constant after the formation of the γ' phase.

Supplementary Note 7. Ex-situ hydrothermal synthesis of MnOx.

Parallel ex-situ experiments in conventional autoclaves confirmed the end products produced in-situ. The following paragraph provides the preparation method for the ex-situ runs. For $P_{K=0.33}$ and $P_{K=0.2}$, $KMnO_4$ was added to 15 mL 0.2 M HNO_3 solution and fully dissolved (5 mmol $KMnO_4$ for $P_{K=0.33}$, and 3 mmol $KMnO_4$ for $P_{K=0.2}$). Then, the solution was transferred into a 25 mL Teflon-lined container and sealed in a stainless steel autoclave (Parr Instrument). The autoclave was then heated in an oven at 160 °C for 24 hr. For $P_{K=0}$, 0.25 M $KMnO_4$(aq) was passed slowly through a column filled with Amberlite® 120 ion-exchange resin in H^+ form to replace the K^+ ions with H^+ [12]. After ion-exchange, [K^+] in the solution was negligible and [Mn] was reduced to ~ 0.08 M, as confirmed by inductively coupled plasma (ICP). Then, 15 mL of the ion-exchanged $KMnO_4$ solution was transferred into a 25 mL Teflon-lined container and then heated in the same condition as $P_{K=0.33}$ and $P_{K=0.2}$. After the thermal treatment, the end products synthesized ex-situ were examined by X-ray diffraction and compared with the in-situ results, as shown in Supplementary Figure 4.

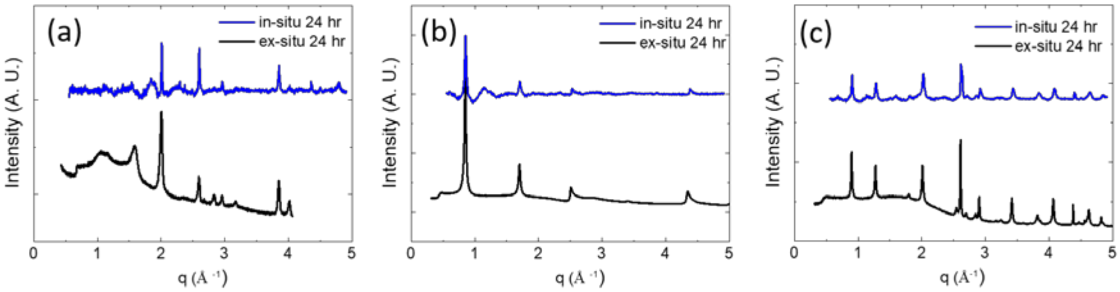

Supplementary Figure 4. X-ray scattering data of (a) $P_{K=0}$, (b) $P_{K=0.33}$, and (c) $P_{K=0.2}$ at 24 hr ex-situ and *in-situ*, showing the end phases are β, δ, and α- MnO_2, respectively. The results show consistency between ex-situ and in-situ runs. The background of the in-situ profiles were manually removed by using GSAS-II software.

Supplementary Note 8. ICP measurements of [K] and [Mn].

Inductive coupled plasma (ICP) measurements were used to determine a. K ion left in the ion-exchanged $KMnO_4$ solution, and b. K stoichiometry in the ex-situ synthesized δ' and α-MnO_2. To fully dissolve the sample, designated amounts of a. or b. was etched in drops of aqua regia for 3 days. The etched solutions were then diluted with DI water and measured by ICP. The concentrations of K and Mn are listed in Supplementary Table 4, and the K stoichiometry in K_xMnO_2 can be found in Supplementary Table 5.

Supplementary Table 4. ICP results of K and Mn concentrations in the ion-exchanged 0.25 M $KMnO_4$(aq).

	[Mn] (M)	[K] (M)
Sample 1	0.10	Negligible
Sample 2	0.07	Negligible
Sample 3	0.07	Negligible
Average	0.08 ± 0.02	Negligible

Supplementary Table 5. ICP results of K stoichiometry in α and δ'-MnO_2.

	x
α-K_xMnO_2	0.10 ± 0.01
δ'-K_xMnO_2	0.31 ± 0.02

Supplementary Note 9. hkl indexing of the δ' phase.

Supplementary Figure 5 explains how the scattering peaks at q = 2.58 and 4.45 $Å^{-1}$ for the δ' phase is indexed. The same d-spacing can be represented by two sets of indexes due to the symmetry of the hexagonally arranged Mn atoms in the δ' phase.

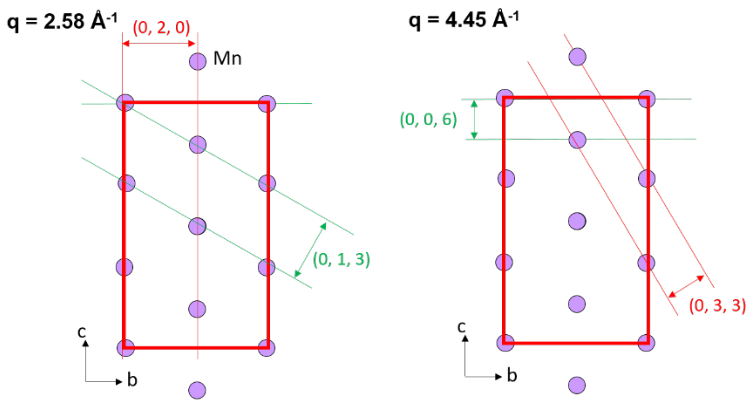

Supplementary Figure 5. Sets of planes that correspond to diffraction peaks at q = 2.58 (left) and 4.45 $Å^{-1}$ (right) of the δ' phase. The unit cell is represented by the red rectangular.

Supplementary Note 10. Formation Energies of Pristine and K-intercalated bulk MnO_2 polymorphs. Methodology details on the bulk structural preparation and DFT Gibbs formation energy calculations of MnO_2 polymorphs in their pristine and off-stoichiometric structures were published previously in Kitchaev *et al.*[13]. In brief, DFT calculations were performed using the Vienna Ab Initio Simulation Package (VASP) with projector-augmented wave (PAW) pseudopotentials, a reciprocal space discretization of at least 25 $Å^{-1}$, and using the SCAN meta-GGA exchange-correlation functional[14], which accurately reproduces the relative energies of MnO_2 polymorphs[8]. Full k-point relaxations were converged to 2×10^{-7} eV/atom on the electronic structure, and a maximum force of 0.02 eV/Å on all atoms. All structures were initialized using the ground state antiferromagnetic (AFM) or ferromagnetic (FM) orderings. K^+ ions were inserted into the 2a, 2b, 8h and 8h' Wyckoff sites in α-MnO_2, and into the octahedral sites in δ. We include a constant vibrational free energy contribution of -0.063 eV/mol to all A_xMnO_2 solids based on the excess entropic contribution to the free energy of β-MnO_2 assumed to be associated with phonons (reference formation Gibbs free energy of -4.837 eV/mol and enthalpy of -5.408 eV/mol taken from thermochemical tables from Hem *et al.*[15] and Kubaschewski *et al.*[16] respectively, entropy of Mn + O_2 reference states ~2.1 meV/mol K). The differences in vibrational free energies within a representative sample of A_xMnO_2 phases were previously calculated in Reference 1 to be small enough not to affect the relative free-energies of the A_xMnO_2 phases, justifying the use of this single constant free-energy term. Finally, to account for the significant self-interaction error inherent to the SCAN functional and the associated error in Mn redox potential, we add a correction to Mn^{2+}, Mn^{3+} and Mn^{4+} - containing structures based on the Mn oxidation state. The correction term is equal to the error in the computed formation enthalpy of $Mn^{2+}O$, $Mn^{3+}{}_2O_3$ and β-$Mn^{4+}O_2$ per Mn atom (+0.38 eV/Mn^{2+}, +0.96 eV/Mn^{3+}, +1.52 eV/Mn^{4+}). This correction is mathematically equivalent to referencing, for example, $K_{0.5}Mn^{3.5+}O_2$ to the experimental formation energies of β-$Mn^{4+}O_2$, $Mn^{3+}{}_2O_3$, K_2O, and K rather than directly to the elemental phases.

Supplementary Table 6. DFT-SCAN computed Gibbs formation free energies of K-Mn-O-H phases in eV/mol, accounting for all referencing schemes and correction terms described in the text. Numbers in bold correspond to the energy we use in our analysis.

**Phase**	**DFT-SCAN ΔG_f at 25°C**	**Experimental ΔG_f at 25°C**	**Experimental Reference**	**$\Delta G_{f,\ DFT\text{-}Expt}$** (eV/mol)
MnO		**-3.762**	Hem 1963[15]	
$Mn(OH)_2$	**-6.198**	**-6.403**	Hem 1983[17]	-0.205
Mn_3O_4		**-13.346**	Hem 1983	
α-Mn_2O_3		**-9.132**	Hem 1978[15]	
γ-MnOOH	**-5.964**	-5.780	Hem 1983	-0.184
R-MnO_2	**-4.783**	-4.789	Fritsch 1997[18]	-0.006
β-MnO_2		**-4.837**	Hem 1983	
α-MnO_2	**-4.767**			
α-$K_{0.0625}MnO_2$	**-5.030**			
α-$K_{0.11}MnO_{1.94}$		-5.60	Birkner 2017[19]	
α-$K_{0.125}MnO_2$	**-5.364**			
α-$K_{0.166}MnO_2$	**-5.520**			
α-$K_{0.25}MnO_2$	**-5.764**			
δ-MnO_2	**-4.558**			
δ-$K_{0.21}MnO_{1.87}$		-6.02	Birkner 2017	
δ-$K_{0.33}MnO_2$ (hydrated)	**-5.988**			
δ-$K_{0.5}MnO_2$ (hydrated)	**-6.469**			
δ-$K_{0.75}MnO_2$	**-6.894**			
$KMnO_2$	**-7.313**			

For the phases where experimental data is available, DFT-SCAN together with our referencing and correction schemes computes Gibbs formation free energies with reasonable agreement with experiment, with a mean absolute error of 30 meV/atom, well within the bounds of what could be expected from modern density functional theory. Importantly however, SCAN yields a very small error in the relative formation energy of chemically similar phases, such as for the case of β-MnO_2 and R-MnO_2, suggesting that it is a reliable method to study structure selection.

A recent calorimetry study directly measured the Gibbs free energy of K-intercalated MnO_2 polymorphs, extrapolated from melt-drop solution calorimetry of α- and δ-MnO_2 nanoparticles. These experimental formation energies are slightly more exothermic than our DFT-SCAN computed values, but the measured stoichiometries are also slightly oxygen-deficient compared to our computed structure models, which can be a source of the disagreement. In our computed structural models, we use a self-consistent set of structures with K_xMnO_2 stoichiometries, meaning that calculated trends within our structure set should be an accurate model representation of the experimental compounds.

To evaluate free-energies in solid-aqueous equilibria, we use a modified empirical aqueous ion energy referencing scheme similar in construction to Persson *et al.*[20], but adapted from DFT-PBE solid formation energies to DFT-SCAN formation energies. We set the Gibbs free energy of water to -2.468 eV/mol, derived by equating the enthalpy of water vapor to that of a computed isolated water molecule, and then

using the standard free energy of condensation to obtain the free energy of liquid water. Similarly, we use the isolated H_2 and O_2 molecules to compute the enthalpy of these molecular reference states, and rely on their standard entropies to set their Gibbs free energies (-0.403 eV/mol for H_2, -0.633 eV/mol for O_2). The Gibbs free energy of the aqueous ions are similarly derived using their standard electrochemical potentials, on top of the computed enthalpy of their elemental reference states. The resulting aqueous ion energies are shown in Supplementary Table 7.

Supplementary Table 7. Referenced aqueous ion free energies, in eV/atom.

**Aqueous Ion**	**ΔG_f at 25°C 1 Molarity**
H^+ (aq)	0.000
Mn^{2+} (aq)	-2.363
MnO_4^- (aq)	-4.634
K^+ (aq)	-2.926

Combining the referenced aqueous ion formation energies with the DFT-SCAN solid phase formation energies, we construct calculated Pourbaix Diagrams for the Mn-H_2O system, at 25°C, [Mn] = 10^{-6} M, and compare against the experimental Pourbaix diagram. The *E-p*H phase boundaries between the DFT and experimental Pourbaix diagram are in reasonable agreement, with the exception of the γ-MnOOH phase, which DFT predicts to be more stable in water than Mn_2O_3. Although this result is in disagreement with experiment, our prediction actually corroborates two other DFT studies that find γ-MnOOH to be more stable than Mn_2O_3[20,21]. Because this study focuses on the aqueous stability region at pH = 2, E = 1.2 V, this discrepancy does not influence the results of this work.

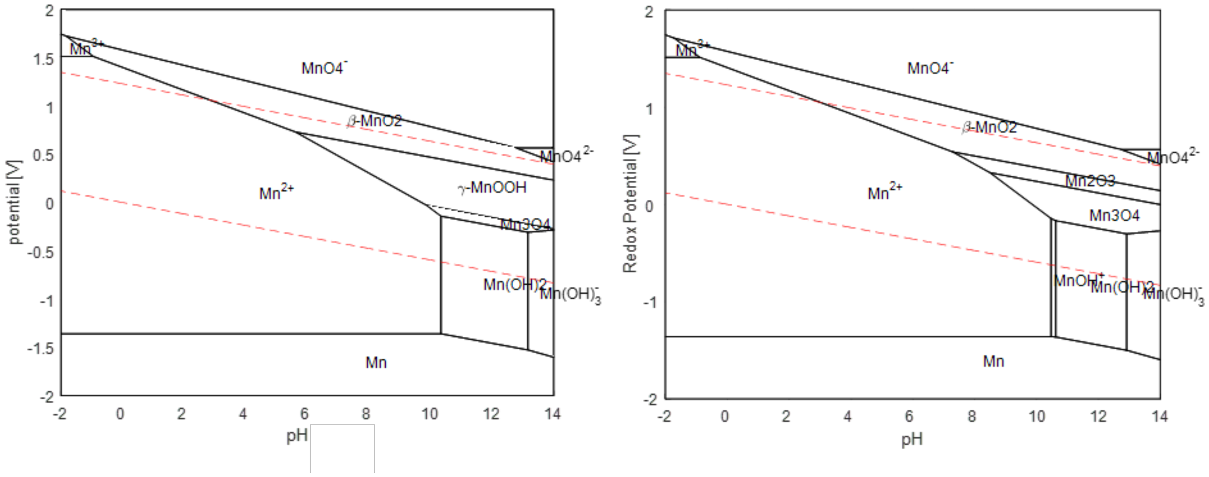

Supplementary Figure 6. Pourbaix Diagrams for Mn-H_2O system, as calculated using DFT formation energies (Left) and experimental formation energies (Right). For both of the figures, the red line in the bottom represents the reversible hydrogen electrode, and that on the top is the reversible oxygen electrode.

Supplementary Note 11. Surface energies of MnO_2 polymorphs and approximation of surface energies for K_xMnO_2 phases

Methodology details on the calculation of surface energies of the MnO_2 polymorphs were published previously in Sun *et al.*[22] Briefly, surface energies are calculated using the SCAN metaGGA functional in density functional theory, using surface slabs generated using the efficient creation and convergence scheme developed by Sun and Ceder[23]. For each conventional bulk unit cell, low-index surfaces are enumerated, and surface energies are calculated within the SCAN metaGGA functional. Surface energy calculations were performed on surface slabs at least 15 Å thick and with 16 Å vacuum.

To compute hydrated surface energies, we attempted to model the first monolayer of strongly-bound water by adsorbing explicit water molecules onto the various surfaces. We previously used this technique to successfully model the hydrated surface energies in $CaCO_3$[24] and FeS_2[25] . However, when we attempt this approach on the manganese oxides, the water molecule dissociates on the surfaces of some MnO_2, but not consistently, nor in a systematic manner, suggesting the presence of a complex interplay between MnO_2 surface geometry, the thermodynamics of associative and dissociative H_2O adsorption, and configurational relaxations of hydrated MnO_2 surfaces. Resolving this interplay connects to the broad question of water splitting catalysis using manganese oxides and falls outside the scope of this work. Nonetheless, we are able to estimate the thermodynamics of MnO_2 surfaces in an aqueous environment, and validate our approximations by comparison to calorimetry results.

The thermodynamic effect of water hydration is to passivate broken bonds, resulting in a reduction of surface energy. The systematic underbinding of bonds in the generalized-gradient approximation often leads to surface energies being consistently under-predicted from experiment by 30%[26], which is approximately the same amount as due to hydration. The dry DFT-calculated surface energies are found to be within the error bars of the hydrated surface energies for bixbyite Mn_2O_3 (γ_{DFT} = 1.36 J/m^2, γ_{Expt} = 1.29 ±0.1 J/m^2) and Pyrolusite β-MnO_2 (γ_{DFT} = 1.54 J/m^2, γ_{Expt} = 1.64 ± 0.1 J/m^2), as measured by Birkner and Navrotsky[27]. Therefore, we use the dry surface energies from SCAN to approximate the quantitative value of the hydrated surface energies.

DFT calculations were performed using the Vienna Ab-Initio Software Package (VASP)[28]. We used the projector augmented wave (PAW) method with the SCAN[14] functional. Plane-wave basis cutoff energies were set at 520 eV for all calculations. Brillouin Zones were sampled using Gaussian smearing, with at least 1000 *k*-points per reciprocal atom for bulk unit cells, and at least 700 *k*-points per reciprocal atom for surface slabs. Atoms were initially relaxed until forces were 0.02 eV/Å. All structure preparations were performed using the Python Materials Genomic (Pymatgen) package[29].

Equilibrium particle morphologies are computed using the Wulff construction. The surface energy term of the size-dependent Pourbaix potential is

$$\left(\frac{1}{R}\right)\frac{\eta\rho\gamma}{N_M} \quad \ldots\ldots.(4)$$

Where η is the shape factor, ρ is the molar volume, and γ is the surface energy. We use a surface energy that is averaged over the particle morphology, computed by the equation

$$\gamma_{\text{Avg}} = \frac{\sum_{hkl} \gamma_{hkl} A_{hkl}}{\sum_{hkl} A_{hkl}} \ldots\ldots(5)$$

The shape factor, η, is unitless and size-invariant, and is computed from Area/Volume constant of the Wulff construction, which is 3 for a sphere [from $(4\pi r^2) / ((4/3)\pi r^3)$]. For each structure, we also provide the surface energies of some facets that are slightly too high in energy to appear on the particle morphology, but could potentially be stabilized by adsorbates or variations in solution conditions.

In our previous work, we computed the surface energies of β-MnO_2, R-MnO_2, which we show here again, for reader's reference. We also include here new surface energy calculations for δ-MnO_2 and α-MnO_2. Please see Supplementary Figure 7 – 10 and Supplementary Table 8 – 12 for numerical details.

Surface energies for α-MnO_2 are given on pristine surfaces, with no K-intercalation. This is a reasonable approximation for relatively dilute K-intercalations, such as the α-$K_{0.125}MnO_2$ phase relevant to this study. This approximation is reasonable because K-intercalation changes the redox of some manganese ions from Mn^{4+} to Mn^{3+}, which reduces the surface energy[19]. However, from a simple broken bond model, the surface energy is proportional to the formation enthalpy, meaning the increased lattice stability upon K-intercalation should slightly increase the surface energy. These two contra-indicated effects suggest that the pristine α-MnO_2 surfaces should be a reasonable approximation for α-$K_{0.125}MnO_2$.

To approximate the contribution of surface energy to the size-dependent Pourbaix free energy of the layered δ-K_xMnO_2-yH_2O phases, we rely on an interpolation derived from δ-MnO_2 and δ-MnOOH, which we resolved previously. Within a broken-bond approximation, the surface energy of a δ-K_xMnO_2-yH_2O structure is dominated by the removal of weak interlayer interactions, with some contribution of cleaving the MnO_2 layer itself. The strength of Mn-O bonds scales with the Mn oxidation state. The strength of interlayer interaction is determined by the fraction of the layer occupied by cations forming ionic bonds spanning the MnO_2 layers, versus weak van der Waals interactions between the MnO_2 layers themselves. As the strength of both the inter- and intra- layer interactions to first order only depend on the quantity of monovalent cations in the layer, we approximate the surface-energy term in the Pourbaix potential $\frac{\eta\rho\gamma}{N_M}$ for δ-K_xMnO_2-yH_2O as a function of x by a linear combination of the surface-energy term derived for δ-MnO_2 and δ-MnOOH, weighted by the quantity of cations or equivalently, the average Mn oxidation state. We then infer the surface energies γ of the δ-K_xMnO_2-yH_2O phases based on their density and approximate shape factor, which is also interpolated from the δ-H_xMnO_2 phases.

While the numerous approximations involved in the computation of the K_xMnO_2 surface energies are certainly not quantitatively precise, the most basic behavior of surface energies in this system is determined by the type of bonds that are broken within each structure – strong metal-oxygen bonds in the case of the 3D-bonded α-$K_{0.125}MnO_2$ versus weak van-der-Waals bonds in the case of the 2D δ-K_xMnO_2-yH_2O phases. As a result, the surface energies of these two families of structures differ by an order of magnitude, such that even with the rough approximations presented here, the general trends in surface-energy controlled phase stabilization remain valid.

Supplementary Table 8. Calculated surface energies, shape factors, and molar volumes of MnO_xH_y phases.

Composition	Surface Energy J/m^2	Shape Factor (Sphere = 3)	Volume/Metal $Å^3$/Mn atom
β-MnO_2	1.54	3.85	27.5
R-MnO_2	1.33	3.53	29.7
α-K_xMnO2	1.19	5.35	33.8
δ-MnO_2	0.12	12.77	32.9
δ-$K_{0.33}MnO_2$	0.14	9.79	44.4
δ-$K_{0.50}MnO_2$	0.18	8.31	44.1
δ-$K_{0.75}MnO_2$	0.25	6.08	48.0

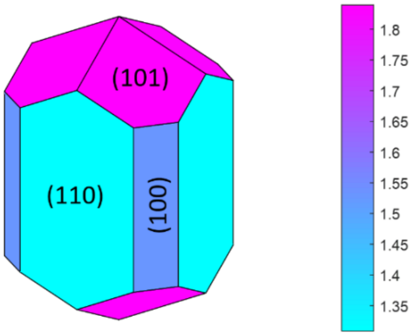

Average surface energy:
1.54 J/m^2
Shape Factor
3.85
Atomic Volume
27.5Å^3/Mn atom

Supplementary Figure 7. Wulff construction of Pyrolusite, β-MnO_2

Supplementary Table 9. Surface energies of Pyrolusite, β-MnO_2

	**Surface Energy (J/m^2)**	**Area Fraction**
110	1.31	0.47
101	1.84	0.38
100	1.53	0.15
**Miller Index**	**Surface Energy (J/m^2)**	**Energy above Wulff (J/m^2)**
001	2.33	0.126

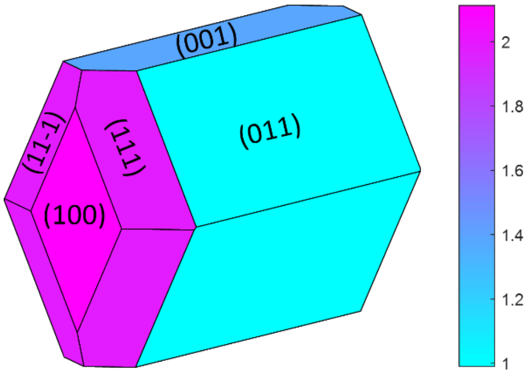

Average SE:
1.33 J/m^2
Shape Factor
3.53
Atomic Volume
29.7Å^3/Mn atom

Supplementary Figure 8. Wulff construction of Ramsdellite, R-MnO_2

Supplementary Table 10. Surface energies of Ramsdellite, R-MnO_2

**Miler Index**	**Surface Energy (J/m^2)**	**Area Fraction**
011	0.99	0.57
001	1.39	0.17
111	1.99	0.11
11-1	2.00	0.1
100	2.11	0.06
**Miller Index**	**Surface Energy (J/m^2)**	**Energy above Wulff (J/m^2)**
110	2.08	0.0146

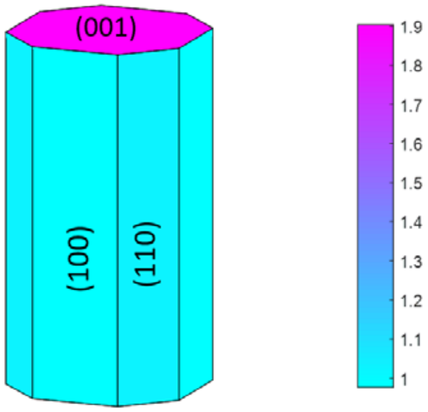

Average SE:
1.19 J/m^2
Shape Factor
5.35
Atomic Volume
33.8 Å^3/Mn atom

Supplementary Figure 9. Wulff construction of Hollandite, α-MnO_2

Supplementary Table 11. Surface energies of Hollandite, α-MnO_2

**Miler Index**	**Surface Energy (J/m^2)**	**Area Fraction**
110	0.98	0.46
100	1.031	0.33
00-1	1.9	0.21
**Miller Index**	**Surface Energy (J/m^2)**	**Energy above Wulff (J/m^2)**
111	2.21	0.0766
11-1	2.21	0.0788

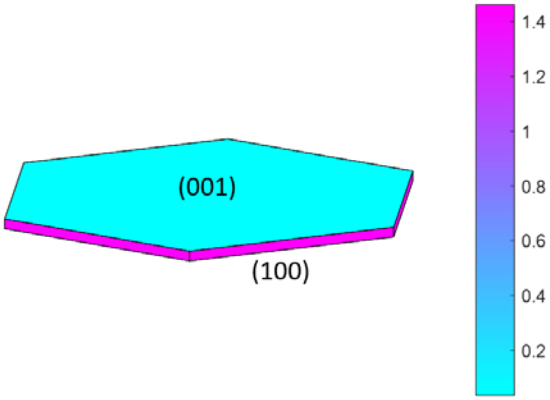

Average SE:
0.11 J/m^2
Shape Factor
12.77
Atomic Volume
32.9 Å^3/Mn atom

Supplementary Figure 10. Wulff construction of Birnessite, δ-MnO_2

Supplementary Table 12. Surface energies of Birnessite, δ-MnO_2

**Miler Index**	**Surface Energy (J/m^2)**	**Area Fraction**
001	0.04	0.95
100	1.46	0.05

S12. δ' phase obtained from $P_{K=0.33}$.

Supplementary Figure 7 shows a selected area electron diffraction pattern image of δ' phase obtained from $P_{K=0.33}$, serving as a further confirmation of the structure of δ'. The image displays a hexagonal-like structure, which is consistent with the Mn sub-lattice that makes up the individual layers of δ' phase. The reflections located on the rings labeled {020} and {013} were measured at 2.50 Å and 2.56 Å, respectively. These reflections are consistent with the d-spacing of the (0,2,0)/(0,1,3) diffraction peak shown in Figure 4(e) in the main text.

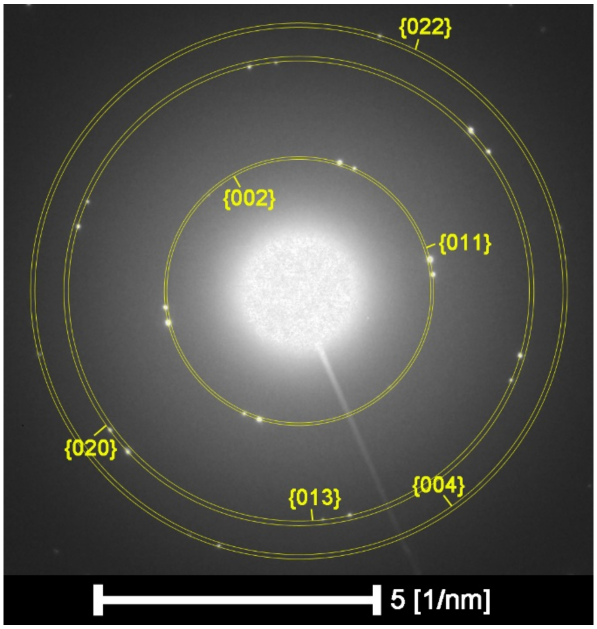

Supplementary Figure 11. Selected area electron diffraction (SAED) patterns of the δ' phase obtained from $P_{K=0.33}$. The double peaks are likely caused by twinning.

Supplementary References

1. Post, J. E. *et al*. Symmetry and Cation Displacements in Hollandites: Structure Refinements ot HoUandite, Cryptomelane and Priderite. *Acta Crystallogr.* **B38,** 1056–1065 (1981).

2. Baur, W. H. Rutile-type compounds. V. Refinement of MnO2 and MgF2. *Acta Crystallogr. Sect. B Struct. Crystallogr. Cryst. Chem.* **32,** 2200–2204 (1976).

3. Post, J. E. Manganese oxide minerals: Crystal structures and economic and environmental significance. *Proc. Natl. Acad. Sci.* **96,** 3447–3454 (1999).

4. Giovanoli, R. Vernadite is random-stacked birnessite. *Miner. Depos.* **15,** 251–253 (1980).

5. Brock, S. L. *et al.* Particle Size Control and Self-Assembly Processes in Novel Colloids of Nanocrystalline Manganese Oxide. *J. Phys. Chem. B* **103,** 7416–7428 (1999).

6. de Wolff, P. M. Interpretation of some γ-MnO 2 diffraction patterns. *Acta Crystallogr.* **12,** 341–345 (1959).

7. Chabre, Y. & Pannetier, J. Structural and electrochemical properties of the proton / γ-MnO2 system. *Prog. Solid State Chem.* **23,** 1–130 (1995).

8. Kitchaev, D. A. *et al.* Energetics of MnO2 polymorphs in density functional theory. *Phys. Rev. B - Condens. Matter Mater. Phys.* **93,** 1–5 (2016).

9. Warren, B. E. *X-Ray Diffraction*. (Dover Publications, 1990).

10. Weidenthaler, C. Pitfalls in the characterization of nanoporous and nanosized materials. *Nanoscale* **3,** 792 (2011).

11. Toby, B. H. & Von Dreele, R. B. GSAS-II : the genesis of a modern open-source all purpose crystallography software package. *J. Appl. Crystallogr.* **46,** 544–549 (2013).

12. Kappenstein, C. *et al.* Permanganic acid: A novel precursor for the preparation of manganese oxide catalysts. *Prep. Catal. VI Sci. Bases Prep. Heterog. Catal.* 699–706 (1995).

13. Kitchaev, D. A., Dacek, S. T., Sun, W. & Ceder, G. Thermodynamics of Phase Selection in MnO2 Framework Structures through Alkali Intercalation and Hydration. *J. Am. Chem. Soc.* **139,** 2672–2681 (2017).

14. Sun, J., Ruzsinszky, A. & Perdew, J. P. Strongly Constrained and Appropriately Normed Semilocal Density Functional. *Phys. Rev. Lett.* **115,** 36402 (2015).

15. Hem, J. Chemical equilibria and rates of manganese oxidation - Chemistry of Manganese in Natural Water. *US Geol. Surv. water-supply Pap. 1667-A* 71 (1963).

16. Kubaschewski, O., Alcock, C. B. & Spencer, P. J. *Materials thermochemistry*. (Oxford, 1993).

17. Hem, J. D. & Lind, C. J. Nonequilibrium models for predicting forms of precipitated manganese oxides. *Geochim. Cosmochim. Acta* **47,** 2037–2046 (1983).

18. Navrotsky, A. Energetics of nanoparticle oxides: interplay between surface energy and polymorphismPresented at the ACS Division of Geochemistry Symposium ?The Impact of Nanoparticle Growth and Transformation Processes on Contaminant Geochemical Cycling?, New Orleans, M. *Geochem. Trans.* **4,** 34 (2003).

19. Birkner, N. & Navrotsky, A. Thermodynamics of manganese oxides: Sodium, potassium, and calcium birnessite and cryptomelane. *Proc. Natl. Acad. Sci.* **114,** E1046–E1053 (2017).

20. Persson, K. A., Waldwick, B., Lazic, P. & Ceder, G. Prediction of solid-aqueous equilibria: Scheme to combine first-principles calculations of solids with experimental aqueous states. *Phys. Rev. B - Condens. Matter Mater. Phys.* **85,** 235438 1-12 (2012).

21. Zeng, Z. *et al.* Towards First Principles-Based Prediction of Highly Accurate Electrochemical Pourbaix Diagrams. *J. Phys. Chem. C* **119,** 18177–18187 (2015).

22. Sun, W., Kitchaev, D., Kramer, D. & Ceder, G. Revealing the Metastable Energy Landscape of Manganese Oxide Precipitation. *Under Rev.*

23. Sun, W. & Ceder, G. Efficient creation and convergence of surface slabs. *Surf. Sci.* **617,** 53–59 (2013).

24. Sun, W. *et al.* Nucleation of metastable aragonite CaCO3 in seawater. *Proc. Natl. Acad. Sci.* **112,** E2735–E2735 (2015).

25. Kitchaev, D. A. & Ceder, G. Evaluating structure selection in the hydrothermal growth of FeS2

pyrite and marcasite. *Nat. Commun.* **7,** 13799 (2016).

26. Goniakowski, J., Holender, J. M., Kantorovich, L. N., Gillan, M. J. & White, J. A. The Influence of Gradient Corrections on Bulk and Surface Properties of TiO2 and SnO2. (1995).

27. Birkner, N. & Navrotsky, A. Thermodynamics of manganese oxides: Effects of particle size and hydration on oxidation-reduction equilibria among hausmannite, bixbyite, and pyrolusite. *Am. Mineral.* **97,** 1291–1298 (2012).

28. Kresse, G. & Furthmüller, J. Efficient iterative schemes for *ab initio* total-energy calculations using a plane-wave basis set. *Phys. Rev. B* **54,** 11169–11186 (1996).

29. Ong, S. P. *et al.* Python Materials Genomics (pymatgen): A robust, open-source python library for materials analysis. *Comput. Mater. Sci.* **68,** 314–319 (2013).
