# Supplementary material for: Understanding crystallization pathways leading to manganese oxide polymorph formation
Source: Nat Commun. 2018 Jun 29;9:2553. doi: 10.1038/s41467-018-04917-y (PMC6026189; doi:10.1038/s41467-018-04917-y)
Supplement: Supplementary file 3 — Description of Additional Supplementary Files [file 41467_2018_4917_MOESM3_ESM.pdf]

Description of Additional Supplementary Files

File Name: Supplementary Data 1
Description: Structure file of the refined alpha phase.

File Name: Supplementary Data 2
Description: Structure file of the refined beta phase.

File Name: Supplementary Data 3
Description: Structure file of the refined delta phase.

File Name: Supplementary Data 4
Description: Structure file of the idealized gamma phase.

File Name: Supplementary Data 5
Description: Structure file of the idealized R phase.
